# The Efficacy of Biodegradable Temporising Matrix for Upper Limb Reconstruction: A Systematic Review and Meta-Analysis

**DOI:** 10.7759/cureus.75994

**Published:** 2024-12-19

**Authors:** Mariana Kostova, Thomas D Alexander, Martha De La Cruz Monroy, Himani Murdeshwar, Haris Duvnjak, Harriet C McCance, Ibrahim Natalwala, Shafiq Rahman, Lauren-Jane Fredericks-Bowyer

**Affiliations:** 1 Otolaryngology, Leeds Teaching Hospitals NHS Trust, Leeds, GBR; 2 Otolaryngology, Warrington and Halton Teaching Hospitals NHS Foundation Trust, Warrington, GBR; 3 Plastic Surgery, Leeds Teaching Hospitals NHS Trust, Leeds, GBR; 4 Surgery, Barts Health NHS Trust, London, GBR; 5 Otolaryngology, Wrexham Maelor Hospital, Wrexham, GBR; 6 Plastic Surgery, Northern General Hospital, Sheffield Teaching Hospitals NHS Foundation Trust, Sheffield, GBR; 7 Plastic Surgery, Nottingham University Hospitals NHS Trust, Nottingham, GBR

**Keywords:** biodegradable temporizing matrix, btm, complex upper extremity wound, dermal regeneration matrix, dermal substitute

## Abstract

The objective of this systematic review and meta-analysis is to assess the efficacy of the biodegradable temporising matrix (BTM) (NovoSorb; PolyNovo Biomaterials Pty Ltd, Port Melbourne, Victoria, Australia) in the reconstruction of complex upper extremity wounds. The authors conducted a systematic review and meta-analysis as per the Preferred Reporting Items for Systematic Reviews and Meta‐Analyses (PRISMA) guidelines assessing the efficacy of BTM in complex upper extremity wound reconstruction. The primary outcome measures were successful BTM integration and the proportion of wounds healed. Secondary outcomes analysed were the average time from BTM application to its integration, the proportion of wounds healed by secondary intention, graft take over BTM, as well as the incidence of infection. The rate of complications as well as scarring and outcomes in upper limb function were also evaluated. The inclusion criteria were met by 12 studies consisting of 164 complex upper extremity wounds. Successful BTM integration was reported in 92.1% (p<0.001) of cases, coupled with wound healing achieved in 90% (p<0.001) of cases overall. The average time to integration for BTM was 37.37 days (p<0.001). The average infection rate for upper extremity wounds with BTM application was 8.5% (p<0.001). Satisfactory scarring and functional outcomes were reported in the majority of the studies. The authors conclude that BTM offers good wound healing outcomes for upper extremity reconstruction. The studies analysed indicate good graft take rates and a low infection incidence; however, further prospective randomised studies are required to support the efficacy of BTM compared to other dermal matrices.

## Introduction and background

The selection of an appropriate reconstruction method for hand and upper extremity wounds with exposed bone, tendon, and/or nerve is often challenging. These complex wounds potentially require flap reconstruction (local, regional, or free flap) which can have debilitating sequelae in comorbid patients [1].

The relationship between poor wound outcomes and patient comorbidities, particularly those that affect the healing process such as diabetes mellitus, has been well established. Diabetes, for example, is associated with prolonged wound healing due to circulatory dysfunction at both the microvascular and macrovascular levels, which delays or inhibits healing [2]. Other risk factors, including advanced age, cigarette smoking, intravenous drug use, and stroke, can further exacerbate wound complications, increasing the risk of infection, sepsis, and even amputation [3]. These risk factors not only affect wound healing but are associated with long-lasting functional effects, often necessitating extended hospital admission and additional resources, which may also compromise outcomes for complex reconstruction [4].

Artificial dermal matrices offer a suitable alternative and can provide good aesthetic and functional outcomes without the associated donor site morbidity of free flap reconstruction [5]. One of these matrices is the biodegradable temporising matrix (BTM) (NovoSorb; PolyNovo Biomaterials Pty Ltd, Port Melbourne, Victoria, Australia) which is an artificial dermal template developed in Australia in 2004 and approved by the Australian Therapeutic Goods Administration for the reconstruction of skin defects of any aetiology [6].

BTM is made of a synthetic material comprised of two layers: an underlying 2-mm-thick biodegradable open-cell polyurethane foam and an overlying temporary non-biodegradable polyurethane sealing membrane (the "lamina"). The underlying layer instigates the formation of a neodermis through angiogenesis, while the overlying lamina ensures there is minimal fluid loss from the wound via evaporation and helps to prevent scar contracture. In the second stage, the lamina is removed, and treatment is completed either by skin graft application or by allowing healing by secondary intention [7].

In their study, Schlottmann et al. [8] demonstrated that the utilisation of BTM provides good wound healing outcomes even in the presence of infection of the wound bed as well as satisfactory functional outcomes in challenging anatomical areas. BTM not only can offer good healing and functional outcomes but may also help to avoid further grafting procedures if there is patient preference or poor compliance with planned post-operative care [9-11].

The authors aimed to amalgamate the existing data by conducting a systematic review and meta-analysis on the application of this matrix to upper limb defects.

## Review

Methodology

Methods

This systematic review and meta-analysis was conducted according to the Preferred Reporting Items for Systematic Reviews and Meta-Analyses (PRISMA) statement standards [12].

Eligibility Criteria

All studies reporting on BTM for upper limb wounds including hand defects were included. There were no restrictions on subjects' age, sex, comorbidities, and wound aetiology. Studies reported in languages other than English were excluded from the review process, as well as all case reports and abstracts.

Outcome Measures

The main primary outcome measure was BTM integration, which was defined as the presence of adequate granulation tissue in the wound bed (at least 95% of the wound bed surface) indicated by a change of colour from white/pale to erythematous/red with or without slight loosening of the sealing membrane [8]. In addition to this, the mean percentage take rate of BTM and wound healing were also analysed as primary outcome measures.

Secondary outcomes included time to integration and the proportion of wounds healed by secondary intention compared to wounds that were grafted with a split-thickness skin graft (STSG). Other secondary outcomes analysed were skin graft take rate, skin graft takes proportional to the area of BTM implanted, and prevalence of wound complications such as infection incidence rate and scarring as well as function.

Literature Search Strategy

Two authors, MK and TDA, independently searched the electronic databases of Google Scholar, PubMed, MEDLINE, Cumulated Index in Nursing and Allied Health Literature (CINAHL), and the Cochrane Central Register of Controlled Trials (CENTRAL). The World Health Organization International Clinical Trials Registry Platform, ClinicalTrials.gov, and the International Standard Randomised Controlled Trial Number (ISRCTN) Registry were also searched to identify further work. The last search was conducted on October 4, 2024. The search terms for our intervention of interest consisted of "biodegradable temporising matrix", "BTM", "upper limb", "hand", "defects", "wounds", and "reconstruction". All terms were combined with adjuncts of "and" as well as "or". To extend the screening for eligible articles, the bibliographic lists were also reviewed for relevant studies.

Selection of Studies

Two authors, MK and TDA, independently assessed the titles and abstracts of articles retrieved from the literature. Articles that met the eligibility criteria were selected after their full texts were reviewed. A consultation was obtained from an independent third author, SR, for any discrepancies in study selection.

Data Extraction and Management

A Microsoft Excel data extraction spreadsheet (Microsoft Corporation, Redmond, Washington, United States) was amalgamated that abided with Cochrane's data collection form for intervention reviews. A pilot test was conducted with the spreadsheet extracting data from random articles and adapting it as needed.

Methodological Quality Review and Risk of Bias

The Newcastle-Ottawa Scale (NOS) [13] was used to assess the methodological quality of all non-randomised studies. This scale uses a star scoring system for three different domains: selection, comparability, and outcome. A maximum score of 9 stars can be awarded for each study.

The Grading of Recommendations Assessment, Development, and Evaluation (GRADE) approach is a system for rating the quality of evidence in systematic reviews. The GRADEpro GDT (GRADEpro Guideline Development Tool [Software]. McMaster University and Evidence Prime, 2024. Available from gradepro.org.) was utilised to assess the quality of evidence from the selected studies and the meta-analysis outcomes.

Data Synthesis

The OpenMeta[Analyst] software (Brown University, Providence, Rhode Island, United States) was used to perform data synthesis with all outcomes reported in forest plots at 95% confidence intervals. The untransformed proportion metric was utilised for dichotomous data in a single group, and the mean metric was used for the assessment of continuous data within a single cohort. The odds ratio (OR) was instigated for the comparison of dichotomous data sets in two groups.

Heterogeneity Assessment

Cochran's Q test (χ2) was used to assess heterogeneity among the studies. In order to quantify any inconsistency, as an additional measure, a calculation of I2 was performed. Interpretation of this was guided as follows: 0-25% representing low heterogeneity, 25-75% representing moderate heterogeneity, and 75-100% representing high heterogeneity. The authors adapted a generic inverse variance function to account for scenarios of moderate to high heterogeneity.

Results

Literature Search Results

**Figure 1:**
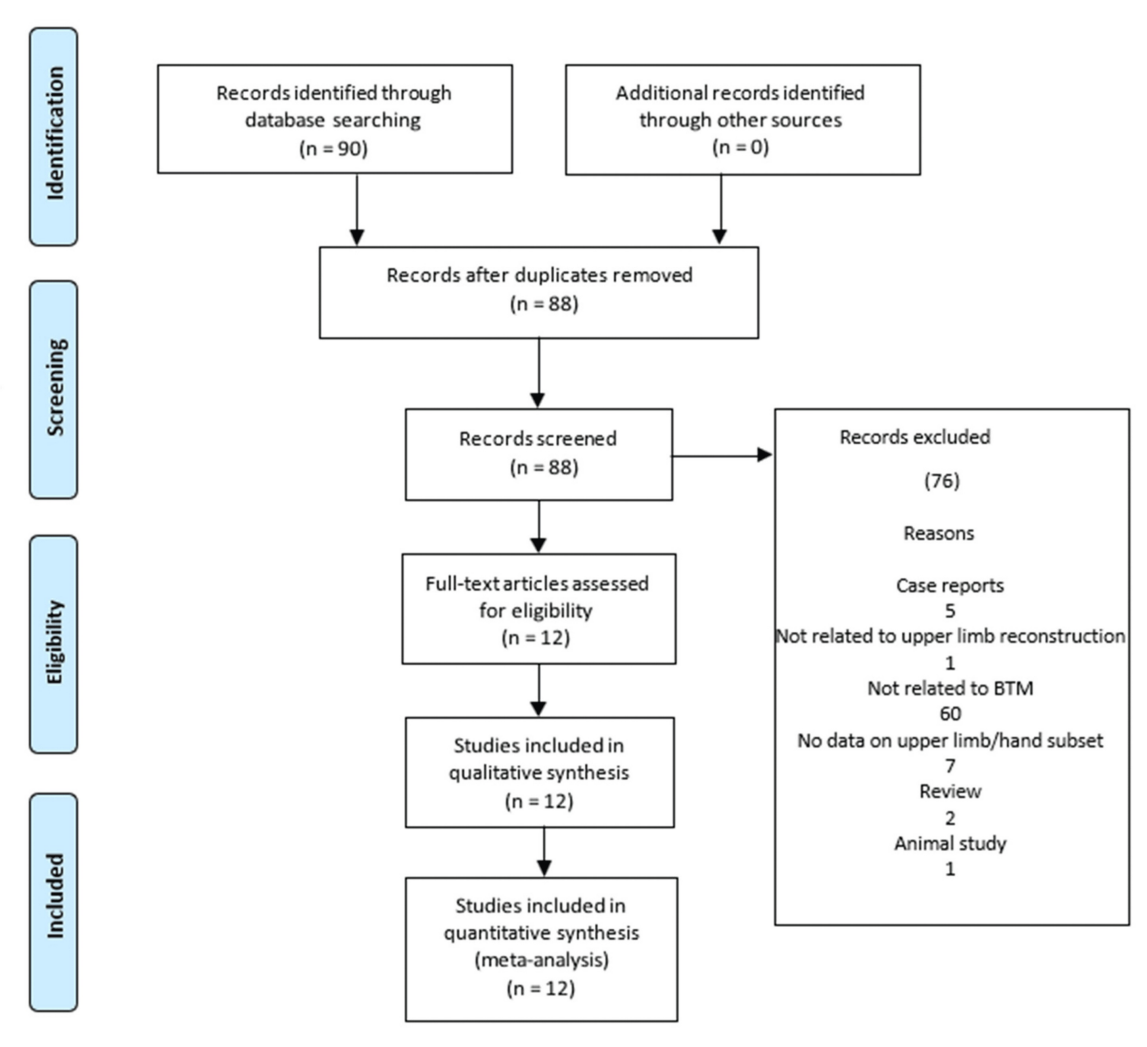
PRISMA flow diagram detailing the search and selection process PRISMA: Preferred Reporting Items for Systematic Reviews and Meta-Analyses; BTM: biodegradable temporising matrix

Eighty-seven articles were identified with 12 meeting the inclusion criteria (Figure 1).

Characteristics of the selected studies are summarised in Table 1. 

**Table 1:** Baseline characteristics of the included studies NR: not reported; SCC: squamous cell carcinoma; RF: radial forearm; UF: ulnar forearm; M: males; F: females; TBSA: total body surface area

Study/year	Study design	Journal	No. of patients	Mean age (years)	Aetiology of wound (trauma (n), infection (n), burn (n), other (n))	Depth of wound (bone, tendon, other soft tissues, etc.)	Average wound size (cm^2^)	Mean follow-up period days/months (range)
Li et al., 2021 [14]	Cohort	ANZ Journal of Surgery	7 (M: 5; F: 2)	66.3	Trauma: 4. Others: 2 (SCC and flap (to cover SCC defect))	Tendon: 6. Paratendon: 1	NR	NR
Cereceda-Monteoliva et al., 2023 [15]	Cohort	European Journal of Plastic Surgery	8 (gender: NR)	51.5	Trauma: 1. Burn: 3. Infection: 2. Others: 2	Tendon: 4. Bone: 4	NR	NR
Concannon et al., 2023 [16]	Retrospective case series	Journal of Burn Care & Research	8 (gender: NR)	49.5	Trauma: 2. Burn: 3. Infection: 2. Others: Ischaemia 1	Tendon: 3. Bone: 5	NR	NR
Fuest and Vögelin, 2024 [17]	Cohort	Journal of Surgery	27 (M: 16; F: 11)	51	Trauma: 20. Infection: 4. Others: 3	NR	Range: 1-200	Median: 36 months (21-111)
Jou and Chepla, 2024 [9]	Retrospective institutional review	Journal of Hand Surgery Global Online	51 (M: 39; F: 12)	44.3	Trauma: 30. Infection: 8. Burn: 12. Iatrogenic: 1	Bone: 24. Tendon: 27	162.5	368 days (51-1216)
Parker et al., 2023 [18]	Retrospective case series	European Journal of Plastic Surgery	2 (M: 2; F: 0)	48.5	Trauma/infection: 1. Burn: 1	Tendon: 1. Paratendon:1	<1% TBSA	NR
Solanki et al., 2020 [10]	Retrospective case series	JPRAS	8 (gender: NR)	50	NR	NR	NR	Median: 3 months (1-7)
Wu-Fienberg et al., 2021 [11]	Cohort	PRS Global Open	6 (M: 4; F: 2)	49.8	Trauma: 6	Bone: 2. Tendon: 6	97	Median: 6 months
Wu et al., 2023 [19]	Cohort	Journal of Hand and Microsurgery	31 (M: 26; F: 5)	45	Trauma: 16. Burn: 4. Iatrogenic/pressure ulcer: 4. Others: 3. Osteomyelitis: 2. Compartment syndrome: 2	NR	100	4.9 months
Najem et al., 2024[20]	Cohort	European Journal of Trauma and Emergency Surgery	5 (gender: NR)	6.5	Avulsion/trauma: 7	NR	180	Median: 17.6 months
Wagstaff et al., June, 2015 [21]	Cohort	ePlasty	7 (gender: NR)	58	Donor site for RF flap	Tendon/paratendon: 7	99.01	Median: 12 months
Wagstaff et al., April, 2015 [22]	Cohort	ePlasty	4 (gender: NR)	59.25	Donor site for RF/UF flaps	Tendon/paratendon: 4	NR	Median: 12 months

Methodological Quality and Risk of Bias Assessment

The NOS [13] was used to assess the quality of the eligible studies, all of which were non-randomised studies. NOS offers a star system for analysis (Table 2). Only one study had a comparative group of patients who had their wounds treated with a different matrix. All the studies reported good-quality selection and outcome reporting.

**Table 2:** NOS to assess the quality of non-randomised studies NOS: Newcastle-Ottawa Scale Rating scale: 7-9 stars: low risk of bias; 4-6 stars: moderate risk of bias; 0-3 stars: high risk of bias One star is awarded for each of the following characteristics: In the Selection domain: (1) adequate definition; (2) case representation; (3) control selection (controls are drawn from the same population as cases); (4) adequate control cases definition. In the Comparability domain, no more than two stars (*) can be given, for how well the study adjusts for confounding factors in the analysis, i.e., comparability of controls and cases. One star is awarded if the study controls for some confounders (such as through matching or statistical adjustment), and two stars are awarded if the study controls for the most important confounders in the analysis. In the Exposure domain: (1) exposure determination; (2) exposure assessment is the same across both groups (exposed and unexposed or cases and controls); (3) non-response rate for case-control studies or loss of follow-up

Paper	Selection	Comparability	Outcome
Li et al., 2021 [14]	**		***
Cereceda-Monteoliva et al., 2023 [15]	**		***
Concannon et al., 2023 [16]	**		***
Fuest and Vögelin, 2024 [17]	**		***
Jou and Chepla, 2024 [9]	**		***
Parker et al., 2023 [18]	**		**
Solanki et al., 2020 [10]	**		***
Wu-Fienberg et al., 2021 [11]	**		***
Wu et al., 2023 [19]	**	**	***
Najem et al., 2024 [20]	**		***
Wagstaff et al., June, 2015 [21]	**		***
Wagstaff et al., April, 2015 [22]	**		***

Primary outcomes

Successful BTM Integration

**Figure 2:**
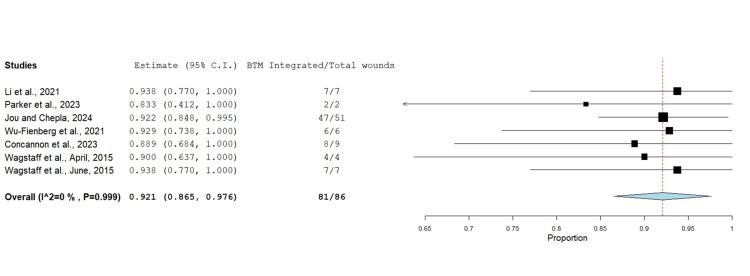
Proportion of wounds demonstrating successful BTM integration (95% and above) within the wound bed using the untransformed proportion metric analysis Overall estimate: 0.921 (95% CI: 0.865, 0.976). Standard error: 0.028; p<0.001 BTM: biodegradable temporising matrix; CI: confidence interval

The proportion of wounds with successful BTM integration (minimum of 95% integration into the wound bed) was measured in seven studies with 86 wounds in total (Figure 2). An untransformed proportion metric analysis demonstrated an integration rate of 92.1% (p<0.001).

Mean Percentage BTM Take

**Figure 3:**
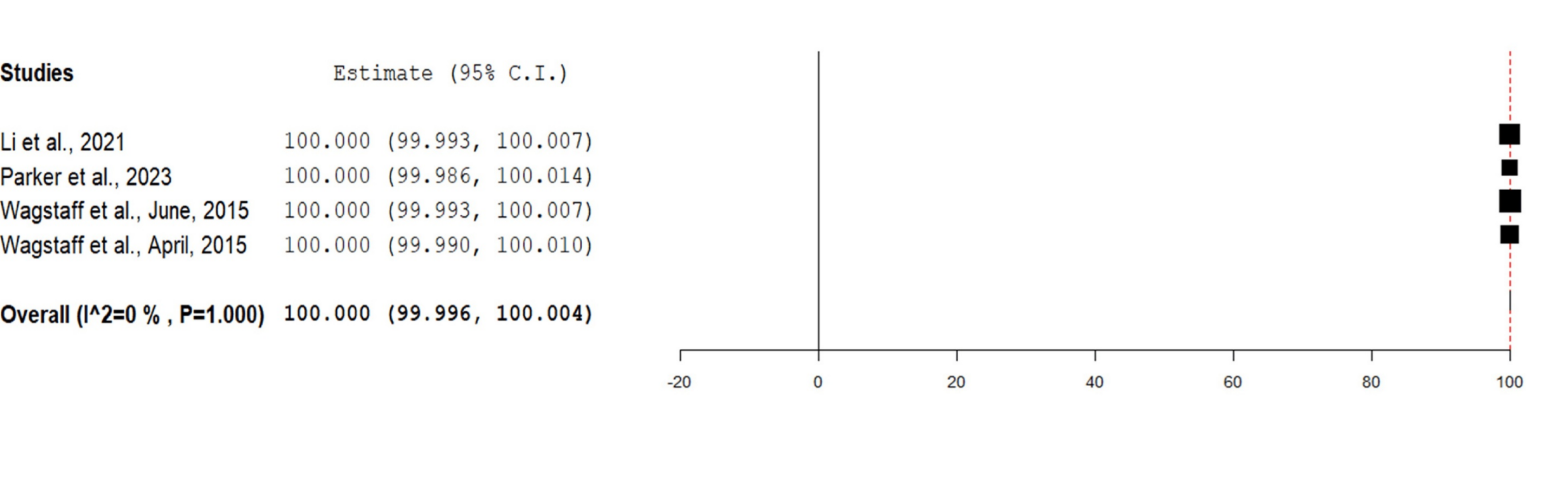
Mean percentage of BTM integrated into the wound bed for each wound using the mean metric analysis Overall estimate: 100 (95% CI: 99.996, 100.004). Standard error: 0.002; p<0.001 BTM: biodegradable temporising matrix; CI: confidence interval

The mean percentage take rate of BTM proportional to the total wound surface area was reported in four studies totalling 20 wounds. All four studies reported 100% mean take rates of BTM for upper limb wounds (Figure 3).

Wound Healing

**Figure 4:**
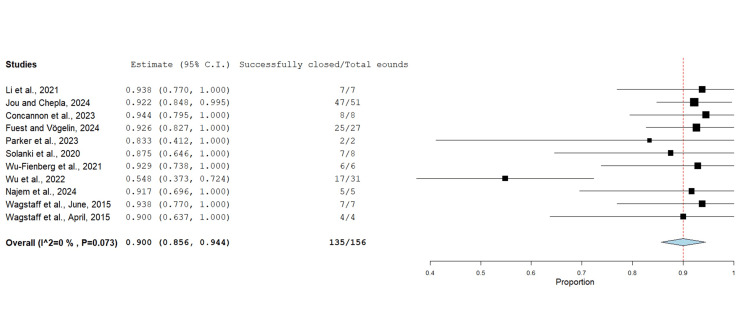
Successful wound closure after BTM using the untransformed proportion metric analysis Overall estimate: 0.900 (95% CI: 0.856, 0.944). Standard error: 0.022; p<0.001 BTM: biodegradable temporising matrix; CI: confidence interval

The rate of successful wound closure overall was reported by 11 studies consisting of 156 cases (Figure 4). In 135 wounds, successful closure by either secondary intention or STSG following BTM integration was obtained at a rate of 90% (Figure 4). Jou and Chepla [9] reported failed closure in four (2.7%) wounds: in two cases, the injury required amputation, and in the other two cases, a secondary flap was performed due to resistant wound infection.

Secondary outcomes

Time to Integration

**Figure 5:**
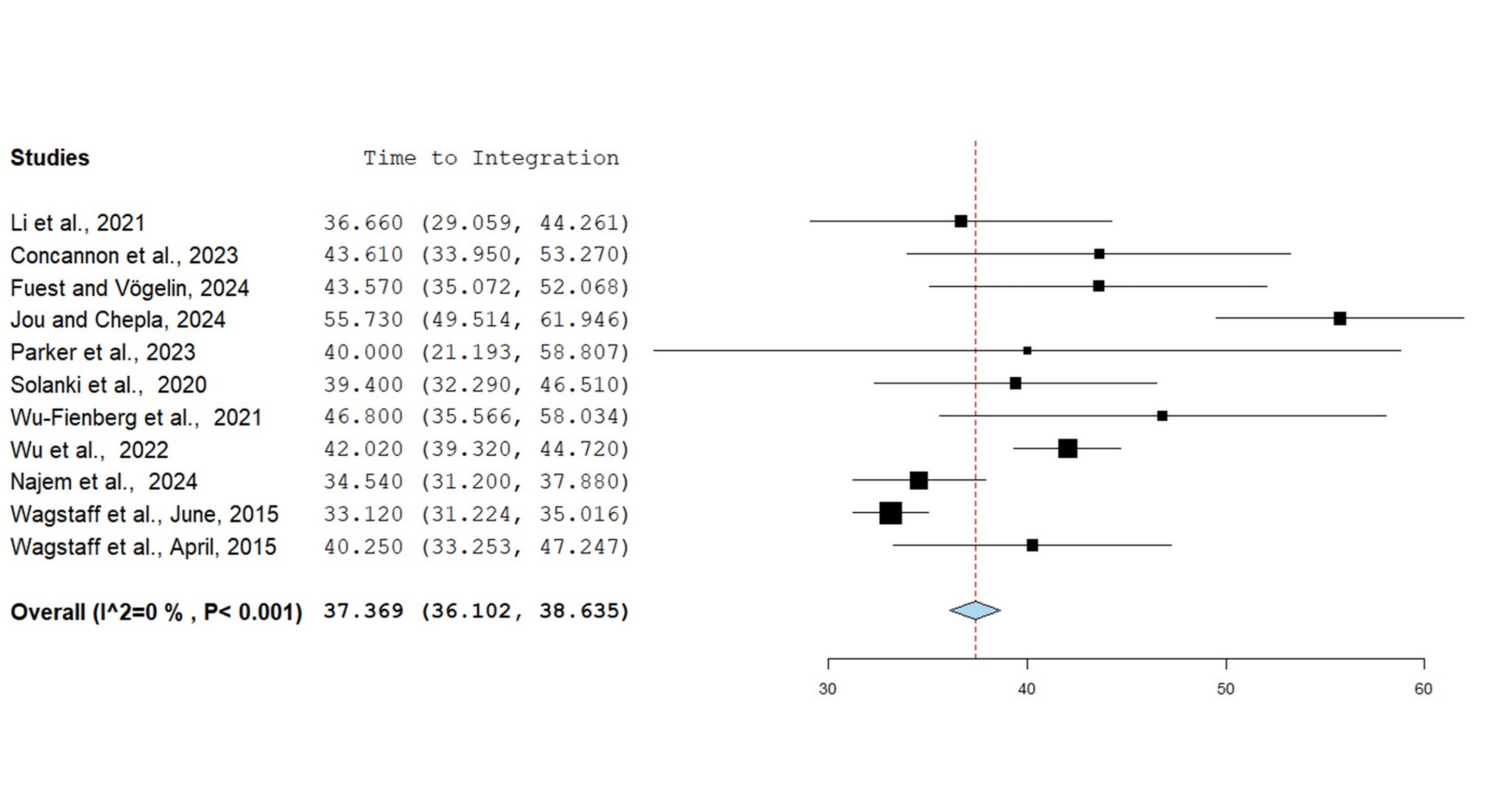
Time from application to integration of BTM in the wound bed (days) using the mean metric analysis Overall estimate: 37.369 (95% CI: 36.102, 38.635). Standard error: 0.646; p<0.001 BTM: biodegradable temporising matrix; CI: confidence interval

Time from application to BTM integration in the wound bed was analysed for 11 studies totalling 156 wounds for upper limb defects (Figure 5). The average number of days required for integration was 37.37 (p<0.001). Most of the studies assessed this time as the days from BTM application to when the wound bed was deemed to be ready for STSG application or in some instances when the wound had already healed.

Healing by Secondary Intention

**Figure 6:**
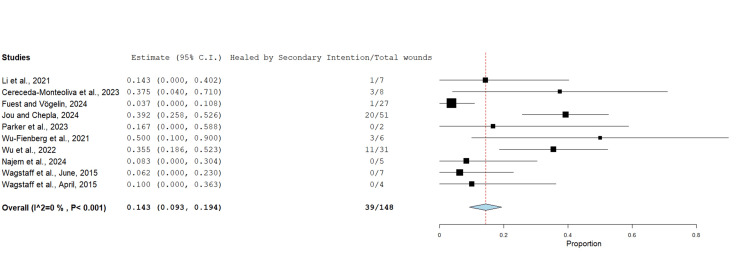
Proportion of wounds left to heal by secondary intention after BTM using the untransformed proportion metric analysis Overall estimate: 0.143 (95% CI: 0.093, 0.194). Standard error: 0.026; p<0.001 BTM: biodegradable temporising matrix; CI: confidence interval

Ten studies in total reported on the incidence of upper limb wounds being left to heal by secondary intention post-application of BTM. Overall, 39 out of 148 wounds were left to heal by secondary intention (Figure 6).

Skin Grafting Versus Healing by Secondary Intention

**Figure 7:**
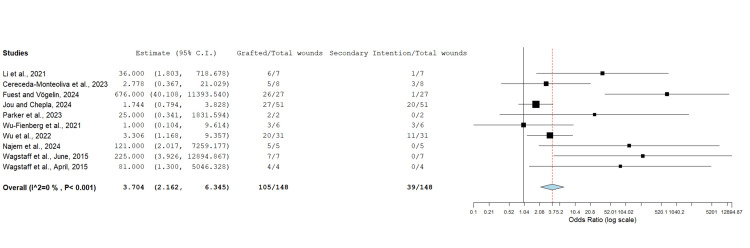
Proportion of wounds undergoing skin grafting versus wounds left to heal by secondary intention Odds ratio: 3.704 (95% CI: 2.162, 6.345); p<0.001 CI: confidence interval

The proportion of wounds where STSG was utilised over the BTM device after integration versus the proportion of wounds left to heal by secondary intention was compared across 148 wounds (Figure 7). In 39 out of 148 (26.4%) cases, wounds were left to heal by secondary intention, whereas, in 105 wounds (70.9%), STSG was utilised after device delamination. An OR assessment (Figure 7) demonstrated a significantly higher proportion of wounds being treated with BTM as a two-stage procedure undergoing STSG application in the second stage.

Healing by secondary intention was preferred over STSG in some cases due to patient choice, non-compliance, or wound healing without the need for grafting. However, it is clearly demonstrated by Jou and Chepla [9] that healing by secondary intention takes significantly longer at an average of 123.3 days compared to 51.7 days with skin grafting. Thus, the majority of surgeons would opt for skin grafting in order to reduce the healing time.

Graft Take

**Figure 8:**
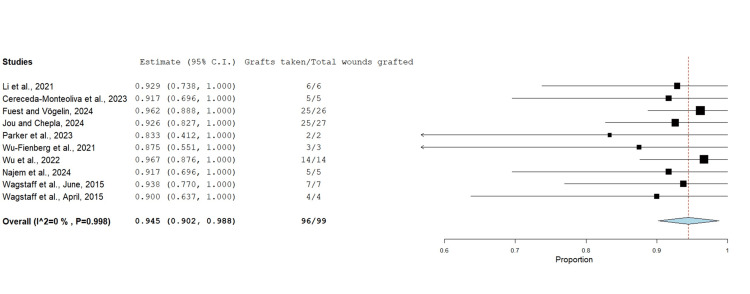
Proportion of grafts taken after BTM delamination using the untransformed proportion metric analysis Overall estimate: 0.945 (95% CI: 0.902, 0.988). Standard error: 0.022; p<0.001 BTM: biodegradable temporising matrix; CI: confidence interval

The proportion of grafts taken after BTM delamination was assessed in 10 studies consisting of 99 wounds (Figure 8). All these studies used STSG and reported excellent results at 94.5%. Only wounds where BTM was seen to have achieved successful integration underwent skin grafting.

Mean Percentage Graft Take Rate

**Figure 9:**
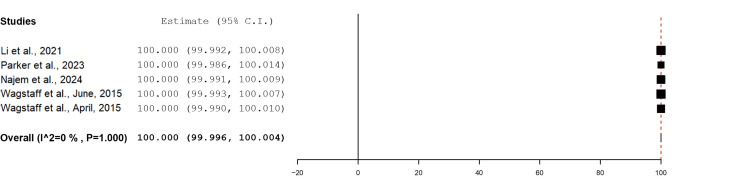
Mean percentage of graft take proportional to the wound bed for each wound using the mean metric analysis Overall estimate: 100 (95$ CI: 99.996, 100.004). Standard error: 0.002; p<0.001 CI: confidence interval

The mean percentage graft take rate proportional to the total wound surface area was reported in five studies with a total of 24 wounds. In all of these studies, a 100% mean graft take rate for upper limb wounds was achieved (Figure 9).

Infection Incidence Rate

**Figure 10:**
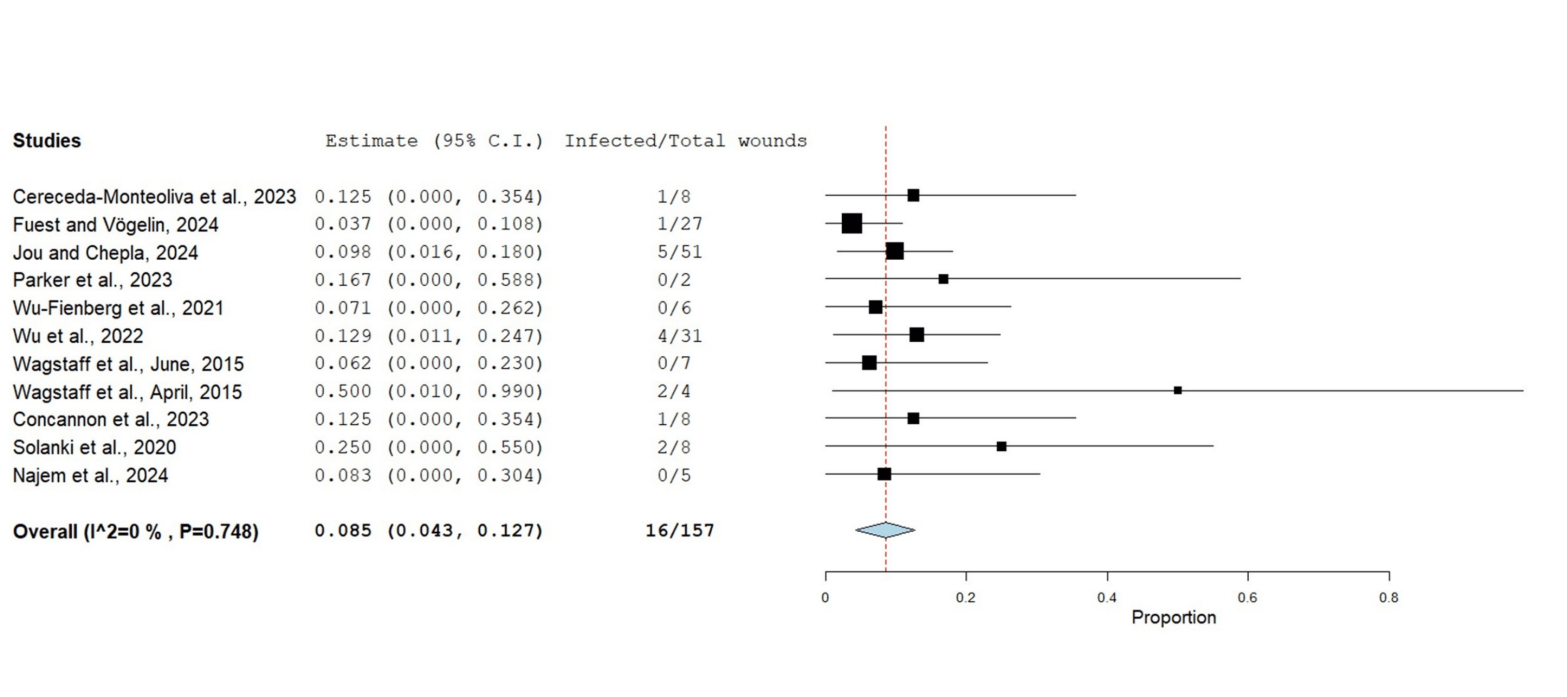
Infection rate after BTM using the untransformed proportion metric analysis Overall estimate: 0.085 (95% CI: 0.043, 0.127). Standard error: 0.022; p<0.001 BTM: biodegradable temporising matrix; CI: confidence interval

The infection rate was reported in 11 studies with a total of 157 cases (Figure 10) at an incidence of 8.5%. This was defined when there was clinical or microbiological evidence of infection at any stage of the BTM integration process. Only Solanki et al. [10] and Wagstaff et al. [22] reported on microorganisms being cultured from wound beds, and among those were *Staphylococcus capitis*, *Streptococcus pyogenes*, *Staphylococcus lugdunensis*, *Staphylococcus aureus*, and *Enterobacter cloacae*.

Other Complications

Other complications such as dehiscence, haematoma, non-adherence, and skin graft complications were reported in six of the studies (Table 3). Jou and Chepla [9] and Wu et al. [19] both reported haematoma formation under the BTM in five (9.8%) and one (3.2%) wounds, respectively. Concannon et al. [16] reported one case requiring tenolysis in upper extremity wounds after the utilisation of BTM. Wu et al. [19] reported two cases (6.5%) of wound dehiscence. Only one study, Fuest and Vögelin [17], reported a skin graft complication where there was insufficient coverage of soft tissue; however, later, this healed on its own.

**Table 3:** Other complications reported by authors n: no of wounds

Study	Wound dehiscence (n)	Haematoma (n)	Non-adherence (n)	Skin graft complications (n)	Tenolysis (n)	Shrinkage (n)	Other (n)
Cereceda-Monteoliva et al., 2023 [15]			1				
Concannon et al., 2023 [16]					1		
Fuest and Vögelin, 2024 [17]				1			
Jou and Chepla, 2024 [9]		5					
Wu et al., 2023 [19]	2	1					1
Najem et al., 2024 [20]						1	

Scarring and Functional Outcomes

Only three studies reported on scar cosmesis overall, employing the Patient and Observer Scar Assessment Scale (POSAS) [23]. This is a reliable scar assessment tool measuring scar quality through a grading system evaluating visual, tactile, and sensory characteristics from both the patient's and clinician's perspectives [14,18,19,23]. Wagstaff et al. [21,22] reported POSAS average scores in both of their papers; however, these were reported as an average for all wounds treated including other sites; hence, it was not possible to evaluate upper extremity wounds separately. Li et al. [14] reported POSAS scores for upper extremity wounds treated with BTM: in the patients' scale, the average score was 2.9±1.099 (SD) and 2.9±0.73 (SD) in the observers' scale, respectively.

Measurable functional outcomes for upper extremity wounds were reported by Fuest and Vögelin [17]. They demonstrated excellent range of movement after BTM application in the metacarpophalangeal (MCP) joints at an average flexion/extension value of 73° and in the proximal interphalangeal (PIP) joints at 75°. Wu et al. [19] also reported good functionality of wounds that healed by secondary intention following BTM integration; however, there was no objective assessment described. Concannon et al. [16] reported an excellent range of motion locally after BTM utilisation in wounds with exposed tendon. Cereceda-Monteoliva et al. [15] noted no impairment of functional outcomes in wounds reconstructed with the use of BTM. Similarly, Najem et al. [20] did not observe any functional impairment in the upper extremity range of motions in all cases after BTM application despite reporting shrinkage in one of the wounds.

Assessment of Quality of Outcomes

The quality of evidence from eligible studies was assessed with GRADEpro GDT. It demonstrated overall low quality of evidence of all of the outcome measures analysed due to the observational design of the selected studies and the inconsistent nature of the research performed (Table 4).

**Table 4:** GRADEpro GDT table assessing the quality of evidence from included studies and summarising the quality of outcomes The Grading of Recommendations Assessment, Development, and Evaluation (GRADE) table was generated with the software GRADEpro GDT (available from gradepro.org) CI: confidence interval; OR: odds ratio; BTM: biodegradable temporising matrix

Certainty assessment							No. of cases		Effect		Certainty	Importance
No. of studies	Study design	Risk of bias	Inconsistency	Indirectness	Imprecision	Other considerations	[intervention]	[comparison]	Relative (95% CI)	Absolute (95% CI)		
Successful BTM integration												
7	Non-randomised studies	Not serious	Not serious	Not serious	Not serious	None	81/86 (94.2%)	0/0	Not estimable		⨁⨁◯◯ Low	Important
Mean percentage BTM take												
4	Non-randomised studies	Not serious	Not serious	Not serious	Not serious	None	20/20 (100%)	0/0	Not estimable		⨁⨁◯◯ Low	Important
Wound healing												
11	Non-randomised studies	Not serious	Not serious	Not serious	Not serious	None	135/156 (86.5%)	0/0	Not estimable		⨁⨁◯◯ Low	Important
Time to integration												
11	Non-randomised studies	Not serious	Not serious	Not serious	Not serious	None	156	0	-	Mean 37.369 higher (36.102 higher to 38.635 higher)	⨁⨁◯◯ Low	Important
Healing by secondary intention												
10	Non-randomised studies	Not serious	Not serious	Not serious	Not serious	None	39/148 (26.4%)	0/0	Not estimable		⨁⨁◯◯ Low	Important
Skin grafting vs. healing by secondary intention												
10	Non-randomised studies	Not serious	Not serious	Not serious	Not serious	None	105/148 (70.9%)	39/148 (26.4%)	OR 3.704 (2.162 to 6.345)	306 more per 1,000 (from 173 more to 431 more)	⨁⨁◯◯ Low	Important
Graft take												
10	Non-randomised studies	Not serious	Not serious	Not serious	Not serious	None	96/99 (97%)	0/0	Not estimable		⨁⨁◯◯ Low	Important
Mean percentage graft take rate												
5	Non-randomised studies	Not serious	Not serious	Not serious	Not serious	None	24/24 (100%)	0/0	Not estimable		⨁⨁◯◯ Low	Important
Infection incidence rate												
11	Non-randomised studies	Not serious	Not serious	Not serious	Not serious	None	16/157	0/0	Not estimable		⨁⨁◯◯ Low	Important

Discussion

Extensive upper extremity wounds and those involving exposed tendon, nerve, and bone pose reconstructive challenges, especially in elderly and comorbid patients as well as those with other significant associated injuries [2,4]. These patient cohorts may not be suited to complex forms of reconstruction, which can potentially lead to debilitating sequelae. The evolution of artificial dermal matrices has provided an important additional tool in the surgeons' armamentarium for treating complex upper extremity defects.

BTM is a fully synthetic dermal substitute made of a 2-mm-thick polyurethane open-cell foam, coated by a non‐biodegradable sealing membrane, aiding complex wound reconstruction [6]. It works as a scaffolding for native dermal regrowth of the wound bed and can also help to temporise extensive wound surface areas while awaiting donor site recovery [7]. 

The use of BTM requires a two-stage procedure [21]. Initially, the synthetic porous matrix undergoes hydrolysis allowing for the re-vascularisation and formation of a neodermis. During this, the sealing membrane plays a crucial role in the healing process preventing water loss, reducing wound contraction, and acting as a barrier to infection [24]. In the second stage, once the matrix has been integrated, the sealing membrane is removed. A STSG can be applied, or the wound can be left to heal by secondary intention [9].

BTM proves to be cost-effective compared to biological dermal matrices due to the low production costs of polyurethane compared to dermal matrices derived from animal products [25]. Moreover, the use of some biological dermal matrices may preclude their use for religious or ethical reasons in certain groups due to being sourced from animals [26]. For example, Integra (LifeSciences Corp., Princeton, New Jersey, United States) is derived from bovine collagen and shark chondroitin 6 sulfate [27]. Synthetic dermal substitute utilisation would be less likely to provoke religious or ethical concerns and so could have a wider application [24].

BTM offers a suitable alternative to complex forms of reconstruction for upper limb defects. Patients who are very morbid, frail, or of advanced age are far from ideal anaesthetic candidates, especially if they have multiple risk factors [3]. This means they are not ideal surgical candidates for complex reconstructions and the longer anaesthetic times associated with these procedures. Performing more extensive surgeries, for example, free flaps, can cause problems with donor site morbidity as well [8,9,19]. 

BTM represents a potential alternative reconstructive option, and this quantitative review of 12 studies found that BTM utilisation for complex upper limb wound reconstruction was associated with favourable wound healing outcomes [14,17,19,21]. Successful wound healing was achieved using BTM in 90% (p<0.001) of upper extremity wounds with an overall successful BTM integration rate of 92.1% (p<0.001).

The average time to integration for BTM was 37.37 days (p<0.001) which is longer compared to other dermal matrices. For example, the time to integration for Integra is reported in the range of 21-33 days in several studies [28-30] and for MatriDerm® (Dr. Otto Suwelack Skin & Health Care AG, Billerbeck, Germany) in the range of 3-4 weeks [31]. Our findings of longer healing times were primarily influenced by the Jou and Chepla [9] study in which a significant proportion of wounds were left to heal by secondary intention. This process resulted in an average reported wound healing time of 123.3 days, compared to 51.7 days with skin grafting.

The associated infection rate for BTM application in upper extremity wounds was calculated as 8.5% (p<0.001), which appears more favourable compared to infection rates reported for Integra (16.3-16.9%) [27,28]. According to Wagstaff et al. [32], this can be explained by the synthetic nature of BTM which does not facilitate bacterial overgrowth and subsequently might lead to an inherent resistance to infection. Furthermore, in 2017, Wagstaff et al. [33] implanted BTM into a large anterior cervical defect after radical debridement of necrotising fasciitis and demonstrated successful implantation, integration, delamination, and STSG application of the wound, once again highlighting BTM's strength in an infected wound bed.

An OR assessment found that a significantly higher proportion of defects were managed with STSG post-BTM integration (OR: 3.704; p<0.001) versus allowing them to heal on their own. Enabling healing by secondary intention avoids the need for secondary surgery and may permit more flexibility in cases of poor patient compliance or poor fitness for surgery; however, successful wound healing was achieved with a higher proportion of wounds being managed with STSG after delamination [9,17,19].

Of the defects managed with STSG, 94.5% (p<0.001) had successful graft take with a mean percentage graft take rate of 100% proportional to the surface area of implanted BTM. These results are comparable to other dermal matrices. Integra has reported graft take rates ranging from 95% to 98% [28-30]. Pelnac (Gunze, Tokyo, Japan) has been referenced in the range of between 53% and 95% [34,35], with MatriDerm ranging from 95% to 96% [36,37].

BTM can be applied to a number of different wound bed types for upper limb defects. Wagstaff et al. [21,22] applied it to radial and ulnar forearm donor sites with exposed tendon and muscle obtaining good healing (100% of 11 wounds across the two studies). Li et al. [14] applied it to six wounds with exposed tendon and one wound with exposed paratendon, all of which successfully healed post-BTM application. Concannon et al. [16] similarly reported a 100% overall healing rate in three wounds with exposed tendon and five wounds with exposed bone. Jou and Chepla [9] reported a 92% successful healing rate (47 out of 51 wounds) in 24 (47%) wounds with exposed bone and 27 (53%) wounds with exposed tendon in the wound bed. Wu-Fienberg et al. [11] also reported wounds with exposed tendon achieved healing in all six cases.

BTM offers excellent functional outcomes for upper extremity reconstruction with Fuest and Vögelin [17] identifying an improvement in the average range of movement for digits. They reported excellent attainment of average ranges of digital motion (MCP: 73°; PIP: 75°) in all patients after complex reconstruction with BTM.

Li et al. [14] reported scarring outcomes after using BTM using the POSAS [23] with an average overall opinion of 5.0 (2.2). These are encouraging results but could not be quantitatively analysed due to the heterogeneous and inconsistent reporting across the other studies this paper has reviewed.

In summary, the authors conducted a systematic review and meta-analysis of 12 studies reporting outcomes of BTM utilisation in complex upper extremity wound reconstruction. The main limitation of this review is the absence of comparator groups in the studies included. Consequently, the studies scored relatively low on the methodology quality assessment under the comparability domain (Table 3). Another limitation of this review was the significant heterogeneity of the studies; however, the authors managed to mitigate this by utilising a random-effects model. Nevertheless, there were 164 wounds of various aetiology analysed. Moving forward, randomised clinical trials are essential to fully appreciate the advantages and disadvantages of BTM utilisation in complex upper extremity wound reconstruction.

## Conclusions

This meta-analysis of 12 studies suggests that BTM has good wound healing outcomes, good graft take rates, and a low infection incidence when compared to other biological dermal matrices. However, there have been no prospective randomised studies to date comparing it to other dermal substitutes. Therefore, the authors recommend further randomised controlled trials to enhance the evidence base to assess the efficacy of BTM in the management of complex upper extremity wound reconstruction.
